# How Does Temporal Blurring Alter Movement Timing?

**DOI:** 10.1523/ENEURO.0496-22.2023

**Published:** 2023-09-11

**Authors:** Dominika Drążyk, Marcus Missal

**Affiliations:** Institute of Neurosciences (IONS), Cognition and System (COSY), Université Catholique de Louvain, Brussels 1200, Belgium

**Keywords:** anticipation, eye movements, hazard rate, temporal cognition, temporal expectation

## Abstract

Subjective uncertainty arises because the estimation of the timing of an event into the future is error prone. This impact of stimulus-bound uncertainty on movement preparation has often been investigated using reaction time tasks where a warning stimulus (WS) predicts the occurrence of a “go” signal. The timing of the “go” signal can be chosen from a particular probability distribution with a given variance or uncertainty. It has been repeatedly shown that reaction times covary with the shape of the used “go” signal distribution. This is interpreted as evidence for temporal preparation. Moreover, the variance of the response time should always increase with the duration of the delay between the WS and the “go” signal. This increasing variance has been interpreted as a consequence of the temporal “blurring” of future events (scalar expectancy). The present paper tested the validity of the temporal “blurring” hypothesis in humans with a simple oculomotor reaction time task where subjective and stimulus-bound uncertainties were increased. Subjective uncertainty about the timing of a “go” signal was increased by lengthening the delay between the WS and the “go” signal. Objective uncertainty was altered by increasing the variance of “go” signal timing. Contrary to temporal blurring hypotheses, the study has shown that increasing the delay between events did not significantly increase movement timing variability. These results suggest that temporal blurring could not be a property of movement timing in an implicit timing context.

## Significance Statement

It is often assumed that the estimation of the timing of a distant future event is more error prone (or variable) than the estimation of a closer one. This phenomenon is referred to as temporal “blurring.” Metaphorically, this could be compared in the spatial domain with the blurring of a visual stimulus when perceived at an increasing distance. Surprisingly, in the current oculomotor implicit timing task, movement variability actually decreased with the increasing temporal delay between a warning and an imperative “go” signal. Therefore, temporal blurring is not a general property but depends on the precise context of timing behavior.

## Introduction

Successfully processing the temporal uncertainty associated with a future event is essential to prepare an appropriate behavioral response. However, the timing of a movement is affected by at least two sources of uncertainty: the intrinsic temporal stochasticity of the stimulus (referred to as “stimulus-bound” or objective uncertainty) and the uncertainty about one’s estimation of its timing (subjective uncertainty). Movement timing is often studied with a paradigm where a warning stimulus (WS; e.g., a spot of light or a sound) precedes the appearance of an imperative “go” signal the subject has to quickly respond to; Woodrow, 1914; Niemi and Näätänen, 1981). The delay between the WS and the “go” signal is referred to as the foreperiod (FP). Before the “go” signal, temporal preparedness increases and reduces movement latency that is commonly used as a dependent variable (e.g., keypress reaction time or eye movement latency). It has been suggested that this process could rely on an estimation of the hazard rate (HR) of the “go” signal that is defined as the conditional probability that the stimulus will appear given that it has not appeared yet (Niemi and Näätänen, 1981; Luce, 1986; Trillenberg et al., 2000; Nobre et al., 2007; Vangkilde et al., 2012, 2013; Herbst et al., 2018; Tomassini et al., 2019; Salet et al., 2022). Mathematically, HR rapidly increases during the FP and reaches a maximum at the time of the longer “go” signal. Therefore, movement latency should be shorter when HR reaches that maximum. The subjective variant of the HR hypothesis further suggests that the inner representation of future events could be “blurred.” Indeed, the estimation of the timing of a distant future event is more error prone (or variable) than the estimation of a closer one. This could be modelled by convolving the objective probability distribution of the “go” signal with a variable Gaussian kernel whose variance linearly increases with elapsed time (Janssen and Shadlen, 2005; Pasquereau and Turner, 2015; Herbst et al., 2018) or equivalent procedure (de Hemptinne et al., 2007). Subjective uncertainty about the precise timing of a future event could explain why the variability of latency increases as the delay between the WS and the “go” signal increases. This phenomenon is probably a particular instantiation of the scalar variability property that describes how temporal perception becomes more variable as the duration to be estimated becomes longer (Gibbon, 1977; Gibbon et al., 1984; Gibbon and Church, 1990; Gallistel and Gibbon, 2000; Wearden and Lejeune, 2008).

An alternative hypothesis to HR suggests that in certain circumstances (e.g., absence of reward) the brain could not perform the delicate computation of HR but could instead use an internal representation of the probability density function of the “go” signal to plan a motor response (referred to as the PDF hypothesis). Where the PDF is maximum (peak value for a symmetrical function) movement latency should be minimum given the higher number of “go” signals experienced by the subject at that time. Stimulus timings infrequently experienced on the sides of the PDF should evoke longer latencies and an inverted U distribution of reaction times is predicted (Grabenhorst et al., 2019, 2021). The PDF hypothesis further suggests a form of probabilistic “blurring” where the size of the blurring kernel does not linearly increase with time but is a function of the probability distribution of the “go” signal. If a Gaussian-shaped distribution of “go” signal timing is used, then movement timing variability should be minimal around the mean value and increase on both sides of the distribution.

Lastly, in contrast with HR and PDF, the formalized multiple trace theory of temporal preparation (fMTP) suggests that a representation of “go” signal timing could emerge by classical associative learning without the need for sophisticated probability computations (Los et al., 2014; Salet et al., 2022). In fMTP, a memory trace of previously experienced timings is used to guide future behavior. For instance, a shorter FP duration during trial “*n*−1” than during the current trial “*n*” will be associated with a relatively shorter movement latency. Temporal blurring is explicitly represented in this model by a “smearing” factor *k*. This aspect of fMTP is functionally equivalent to a convolution with a Gaussian kernel.

The aim of the present study was to further test the hypothesis of temporal blurring in a simple oculomotor reaction time task where the timing of the “go” signal could be constant or vary according to a Gaussian-shaped distribution.

## Materials and Methods

### Subjects and ethics

The present study was conducted at the Université catholique de Louvain. Participants were volunteers (18–65 years old) who did not suffer from known neurologic or psychiatric diseases and did not take psychoactive substances at least a day before the experiment. Participants had corrected to normal vision if necessary. Three participants were excluded from the study because of obvious misunderstanding of the task or extensive noise in the oculomotor signal. This resulted in 42 participants included in the analysis (average ± SD, age 26.50 ± 4.30 years, 23 women). Participants provided their informed written consent to take part in this study. All procedures were conducted in accordance with the Declaration of Helsinki guidelines and approved by the Ethics Committee of the Université catholique de Louvain under number B403201733677. Data are available to participants on written request.

### Experimental design

**Figure 1.**
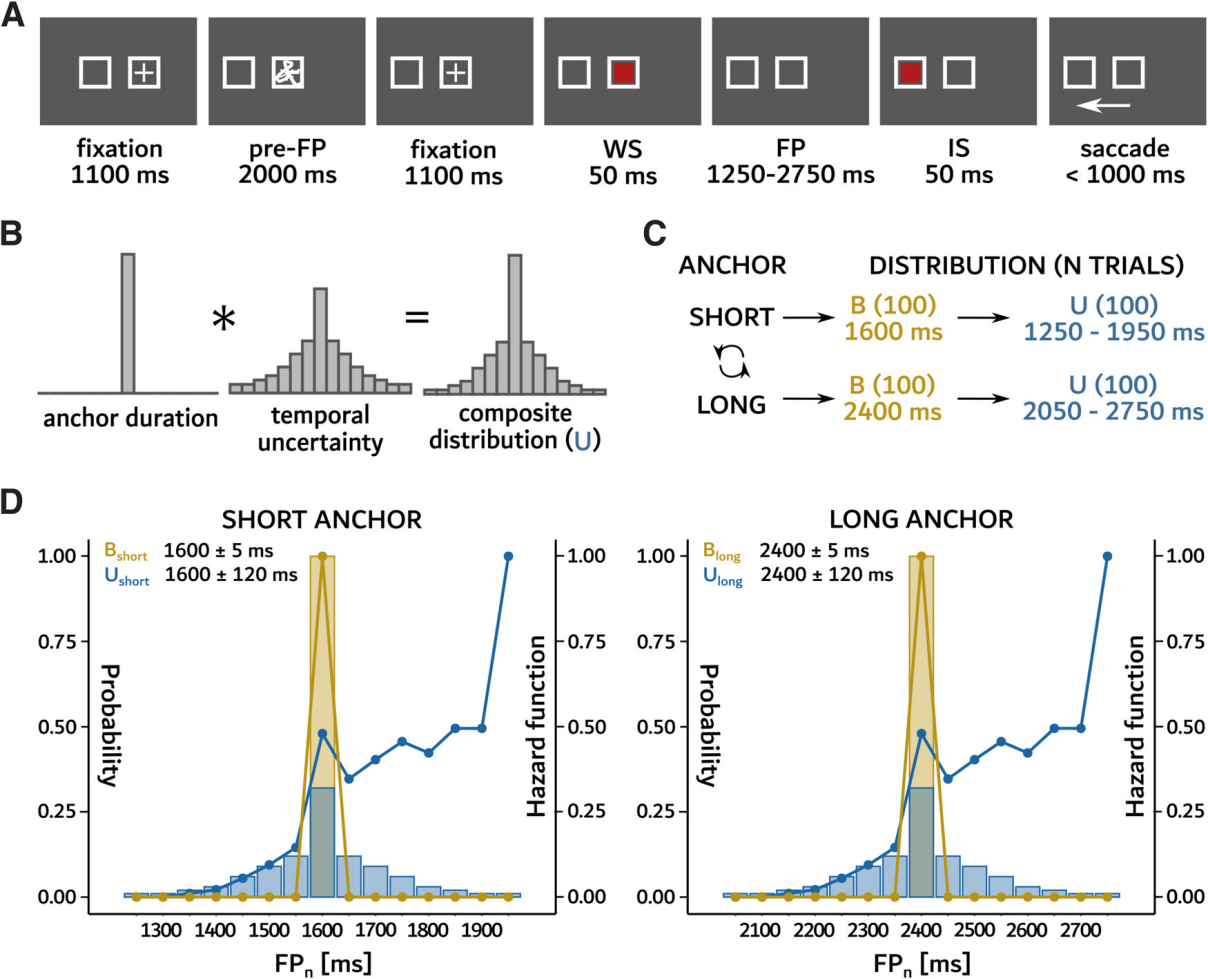
Description of the experimental paradigm. ***A***, Timeline of visual events on the screen in front of the subject. First, two square boxes appeared with a fixation cross (+ sign) in the central one. The location of the eccentric box was randomly assigned to the left or to the right of the central one with the same probability. Afterwards, a noninformative visual stimulus appeared in the central box for 2000 ms (preforeperiod or “pre-FP” interval) followed by another 1100-ms fixation period. Thereafter, the warning stimulus (WS) was briefly presented in the central box (first red square). The appearance of the WS initiated the FP until the imperative stimulus (IS or “go” signal) appeared in the eccentric box (second red square). The arrow illustrates the direction of the correct saccade executed after the IS appearance at the end of the description of the Figure 1*A*. ***B***, The high uncertainty U distribution was a composite obtained by convolving the anchor duration with a Gaussian kernel. ***C***, Data collection started with either the short or the long anchor duration (counterbalanced between subjects). For a given anchor duration, two blocks of 100 trials each were collected: first, a baseline (B) condition consisting of only one duration; second, a block of trials drawn from the U distribution. Number of trials (‘N TRIALS’) per condition is included in the round brackets at the end of the description of the Figure 1*C*. ***D***, Distributions of short (left panel) and long (right panel) anchors, grouped in 50-ms bins. Colored traces represent the B and U hazard functions.

Each trial started with the appearance of two white boxes (3 × 3° visual angle each, see Fig. 1*A*). One box was located in the center of the screen (central box) and the second one was located either to the left or to the right (eccentric box, eccentricity 9.5° of visual angle from the central box) with the same probability (*p* = 0.5). These boxes were used to exclude potentially confounding effects of spatial uncertainty about the future position of the imperative “go” signal. Participants were initially required to maintain visual fixation of the central box for 1100 ms. Afterwards, an abstract noninformative shape was displayed in the central box for 2000 ms (referred to as the “pre-FP” interval). This pre-FP interval was introduced to potentially use the same paradigm with an explicit timing cue (not the aim of the present study). Next, the warning stimulus (WS; red square) was displayed in the central box for 50 ms. After a variable delay (foreperiod or FP, lasting between 1250 and 2750 ms) the imperative “go” stimulus (IS; eccentric red square) was displayed in the eccentric box for 50 ms. Participants were instructed to maintain gaze on the central box during the pre-FP and FP intervals and then initiate a visually-guided saccade toward the IS as quickly as possible. Trials ended with an intertrial interval (ITI) of randomized duration (1050 ± 400 ms).

10.1523/ENEURO.0496-22.2023.f1-1 Extended Data Figure 1-1 Diagnostic plots for the *FP_n_.rs1* model refitted using different distributions. ***A***, *Gaussian* distribution, link: identity. Plots were generated using R package performance (Lüdecke et al, 2021). ***B***, Gaussian distribution, link: log. ***C***, Gaussian distribution, link: identity, fitting the log-transformed data. ***D***, Gamma distribution, link: identity. Plots were generated using R package *DHARMa* (Hartig, 2020). ***E***, Gamma distribution, link: log. ***F***, Inverse Gaussian distribution, link: identity. ***G***, Inverse Gaussian distribution, link: log. Download Figure 1-1, EPS file.

In each block of trials, average FP duration was either 1600 ms (short) or 2400 ms (long), collectively referred to as “anchor.” For each anchor, two blocks of 100 trials each were displayed: first, a block of trials with a constant FP duration referred to as the baseline condition (abbreviated as B distribution, estimated technical jitter σ≈ 5 ms); second, a block of trials drawn from a distribution composed of the same anchor durations (1600 or 2400 ms) but with increased temporal uncertainty (referred to as U distribution, σ = 120 ms). The rationale for using two different anchor durations was to test the “temporal blurring” hypothesis. This hypothesis suggests that the variance of temporal estimations should increase with elapsed time (scalar expectancy; Gibbon, 1977). The U distributions were obtained by convolving anchors with a Gaussian-shaped distribution (Fig. 1*B*). Figure 1*D* shows B and U distributions for both short and long anchors together with their hazard rate (HR). In the computation of HR, the conditional cumulative distribution function (CDF) was used as defined in Salet et al. (2022) to compute the survival rate. The HR function of B is composed of a single peak. The HR function of U has two separated maxima: one local maximum around the anchor value and another one for the longer FP (HR = 1).

In summary, there were four different blocks of trials (average ± SD): *B_short_* (anchor = 1600 ± 5 ms), *U_short_* (anchor = 1600 ± 120 ms), *B_long_* (anchor = 2400 ± 5 ms), and *U_long_* (anchor = 2400 ± 120 ms; see Fig. 1*C*). A block of B trials was always firstly collected with anchors being either 1600 ms or 2400 ms. This block was followed by the corresponding U block of trials. The sequence was then repeated with the other anchor. As an example, if the anchor in the first B block was 2400 ms (*B_long_*), it was followed by a U block of trials with the same anchor duration (2400 ms or *U_long_*). Next, a block of B trials with the other anchor duration (in this example, 1600 ms or *B_short_*) was presented, followed by the corresponding U block of trials (1600 ms or *U_short_*). This design was used to minimize carry-over effects. Indeed, experiencing U before B would change latencies in response to B that would not be a baseline any more (see Los et al., 2017, 2021; Crowe et al., 2021). This carry-over effect could persist even a week after changing the probability distribution of the FP (Mattiesing et al., 2017). Pairs of blocks of different anchors were presented in random order, counterbalanced between subjects.

### Data collection and preprocessing

Eye movements were recorded binocularly at 500 Hz using an EyeLink1000 (SR Research), calibrated at the beginning of each data collection session and recalibrated every 30 trials. Stimuli display and eye movement recordings were created using Experimental Builder (SR Research). Stimuli were displayed on a high-resolution screen (size: 54 × 30 cm, 1920 × 1080 pixels, VPixx Technologies) at 60 Hz. Subjects were sitting at a distance of 80 cm from the display screen. Oculomotor recordings of poor quality were excluded from the analysis using an artifact rejection algorithm (DataViewer, SR Research). All trials including frequent blinking were removed from the analysis. Further, if the continuity of the oculomotor recording was not preserved throughout the whole trial, or if a drift of calibration occurred, it was also excluded. Eye blinks and lost eye signal amounted to 5.07% of all trials (see Table 1). Saccades were detected using an amplitude (>1°), velocity (>22°/s) and acceleration (>3800°/s^2^) criteria. Only visually-guided saccades initiated between 100 and 1000 ms after IS onset and landing within the IS box were further analyzed (69.5% of all trials).

**Table 1:** Data preprocessing number and percentage of the trials that entered the final analysis

Number of trials	*N*	% of total
Total	16,800	100.00
After artifact rejection	15,948	94.93
With visually guided saccades	11,676	69.50

10.1523/ENEURO.0496-22.2023.t1-1 Table 1-1 Linear mixed models random structure selection for the analysis of FPn, FPn-1 HRrec and sequence effects on saccade latency. Models were fitted using the restricted maximum likelihood method. Download Table 1-1, DOC file.

10.1523/ENEURO.0496-22.2023.t1-2 Table 1-2 Linear mixed models random structure selection for the analysis of FPn, FPn-1 HRrec and sequence effects on saccade latency. Models were fitted using the restricted maximum likelihood method. Download Table 1-2, DOC file.

### Statistical analysis, linear mixed effects model

Trial-by-trial saccadic latencies were fitted using a linear mixed models approach (LMM; R package *lme4* library version 1.1–30; Bates et al., 2015). Reaction time data are usually not normally distributed and fitted using non-Gaussian distributions (Baayen and Milin, 2010; Lo and Andrews, 2015). In the present study the latency variable was fitted using various distributions: γ, Gaussian, and inverse Gaussian. A log transformation of saccadic latencies was also calculated and fitted with the Gaussian distribution. All options were examined using the QQplot and residuals (R packages *performance*, Lüdecke et al., 2021 and *DHARMa*, Hartig, 2020). However, none of the above-mentioned distributions improve the fit compared with the Gaussian fit (for the comparison between these different choices, see Extended Data Fig. 1–1). As a result, Gaussian distribution was chosen to fit the latency models. Following the approach of Grabenhorst et al. (2019), we decided to fit three instances of the hazard rate function HR: (1) the classic HR function (*HR_classic_*; see Fig. 1*D*) that presents a general growing trend with increasing FPn duration together with a local peak around the anchor duration; (2) the mirrored HR function (*HR_mirror_*) that is *HR_classic_* mirrored around its mean. *HR_mirror_* shows a general decreasing trend with increasing FPn duration with a local minimum around the anchor duration; (3) the reciprocal HR (*HR_rec_*) that takes the reciprocal of *HR_classic_* (1/*HR_classic_*). *HR_rec_* presents an abruptly decreasing trend with increasing FPn duration and a negligible local minimum around the anchor duration.

Besides the hazard rate variants, main predictors of interest were: *FP_n_*, length of the foreperiod in the given trial *n*; *FP_n_*_– 1_, length of the foreperiod in the previous trial *n*−1; sequence, difference between the length of the current and previously displayed FP (*FP_n_*_– 1_ – *FP_n_*). Additionally, a trial variable (numeric index of the given trial in the block) was introduced in models, following the recommendation of Baayen and Milin (2010). Indeed, it was shown that including a variable representing trial number could help to track the influence of predictors on a trial-by-trial basis.

The labeling of models was as following: the full prefix indicated that the model includes the maximal fixed effect structure incorporating the main predictor and a trial variable. Models including only the main predictor were labeled using the name of the predictor of interest (e.g., *FP_n_*). Further, to describe the random structure of models, the suffix *rs* was used. Different random structures were denoted with consecutive numbers. For example, *FP_n_.rs1* denotes the model with *FP_n_* as an only predictor and the first random slope structure.

The Bayes Information Criterion (BIC; Bolker et al., 2009) was estimated to choose an optimal random term structure for the full models fitted with restricted maximum likelihood method (REML; Zuur et al., 2010; Barr et al., 2013). For all those full models, random intercept structure performed better than the random slope (for details see the values of BIC for models *full.rs1*, *full.rs2*, *full.rs3*, and *full.rs4* for all main predictors and both anchors in Extended Data Table 1-1). Therefore, *rs1* random terms structure was chosen in the further analyses.

Next, to establish the best fixed term structure, models with different fixed terms were refitted using a maximum likelihood method (ML) and compared using BIC. For all main predictors and both anchors, models including only a main effect (*FP_n_.rs1*, *HR_rec_.rs1*, *FP_n– 1_.rs1*, and *sequence.rs1*) performed better than the corresponding full models (for details see the values of BIC in Extended Data Table 1–2). Results show that the incorporation of the *trial* variable in all models did not improve their fits. Therefore, only the main predictor models were refitted using the REML method and presented in Results. For each of the presented models, visual inspection of their diagnostics was conducted to monitor the convergence and accordance with the model assumptions.

**Table 2:** Linear mixed models analysis of *FP_n_* duration effects on saccade latency

Dataset	Model	Fixed terms	β ± SE	95% CI	*p*-value	Random terms	σ [ms]
*U_short_*	*FP_n_*.rs1	Intercept	307.495 ± 14.293	[279.498, 335.467]	<2.22 × 10^–16^	Residual	51.905
		*FP_n_*	−0.054 ± 0.008	[−0.071, −0.038]	1.00 × 10^–10^	Subject	33.571
*U_long_*	*FP_n_*.rs1	Intercept	298.043 ± 20.465	[257.938, 338.114]	<2.22 × 10^–16^	Residual	52.322
		*FP_n_*	−0.027 ± 0.008	[−0.043, −0.011]	9.49 × 10^–4^	Subject	34.545

Models were fitted using the restricted maximum likelihood method. Fixed and random effect structures were chosen in advance (see Extended Data Tables 1-1 and 1-2). Models were fitted using the Gaussian fit (see Extended Data Fig. 1-1 for the comparison between different distribution choices).

σ, SD of the random terms (subject and residual).

### Statistical analysis, simple linear model

Beside the LMM analyses conducted on the trial-by-trial basis, a simple linear model (LM) was used to test specific predictions of the HR, PDF and fMTP hypotheses. This time, the dataset was averaged across subjects and distinct *FP_n_* lengths. Three variants of the HR hypothesis were fitted to the data. Classical HR presented the best fit and therefore was included in Results. Furthermore, the relationship between latency variance and *FP_n_* duration was tested using the slope analysis proposed by Ivry and Hazeltine (Ivry and Hazeltine, 1995; see also Piras and Coull, 2011; Ameqrane et al., 2014). The slope analysis was used to estimate Weber’s fraction *k*, with the assumption that the variability of the subjective time estimation is a constant fraction of the estimated time. This model assumes also that the total response variance comes not only from the timing processes but also from a time-independent source, modelled by the variable *c*. Consequently, if saccadic latencies obey Weber’s law then the data could be modelled using the following equation:

$$\sigma {\left(latency\right)}^{\text{2}}=k^{\text{2}}*FP_{n}{}^{\text{2}}+c,$$

where *FP_n_* represents the length of the foreperiod in the given trial *n* (Ameqrane et al., 2014). As an estimate of effect sizes, adjusted *R*^2^ values are reported (Rosenthal, 1994). In text results are presented using average values and standard errors (average ± SE), unless stated differently. In figures, results are presented as dots (average) and whiskers: dark colored for SE, light colored for 95% confidence intervals (95% CI). The significance level assumed in the present study was 0.01.

### Statistical analysis, equivalence tests

Because of the detection of nonsignificant results in the present study, an equivalence test was performed for the analysis of latency SD between different anchors in the U and B distribution (R package *TOSTER*; Caldwell, 2022) as well as for the influence of the *FP_n_*_–1_ on saccadic latency (R package *parameters*; Lüdecke et al., 2021). This equivalence test evaluates the hypothesis that the effect size obtained for the nonsignificant analyses is small enough to be called negligible (see Lakens et al., 2018 for details). Performing the test requires establishing the smallest effect size of interest (SESOI) that will create the equivalence bounds (eqb). Those should be chosen based on the effect sizes detected in similar experiments from the field. As it was difficult to find a proper sample of experiments mimicking the current design, the experiment of Tomassini et al. (2019) that is the closest to the current paradigm, was chosen as a reference. Reference data included only the control group of Tomassini et al., study. First, the equivalence bounds for the latency SD analysis were calculated based on the critical effect size of Tomassini et al., analyses (eqb = [−1.125, 1.125], Cohen’s *d_z_* type). Then, as the manuscript of Tomassini et al., does not include the analysis of previous FP length effect on response latency, the influence of *FP_n_*_–1_ on the latency was modelled on their data by the authors. Resulting equivalence bounds were based on Cohen’s *f^2^* (Selya et al., 2012) of the calculated model (eqb = [−0.09, 0.09] for the short anchor and eqb = [−0.02, 0.02] for the long anchor).

## Results

To test the influence of the duration of the current foreperiod (*FP_n_*) on saccadic latency in the high uncertainty U distributions, a linear mixed model (LMM) was applied to short (1600 ms) and long (2400 ms) anchors, separately. In short anchor model *FP_n_.rs1* saccadic latency significantly decreased as a function of FP duration showing a potential effect of temporal preparation (β = −0.054 ± 0.008, *p* = 1 × 10^–10^). Similarly, for the long anchor model *FP_n_.rs1* saccadic latency moderately decreased as a function of FP duration (β = −0.027 ± 0.008, *p* = 9 × 10^–4^; see Table 2 for details).

**Figure 2.**
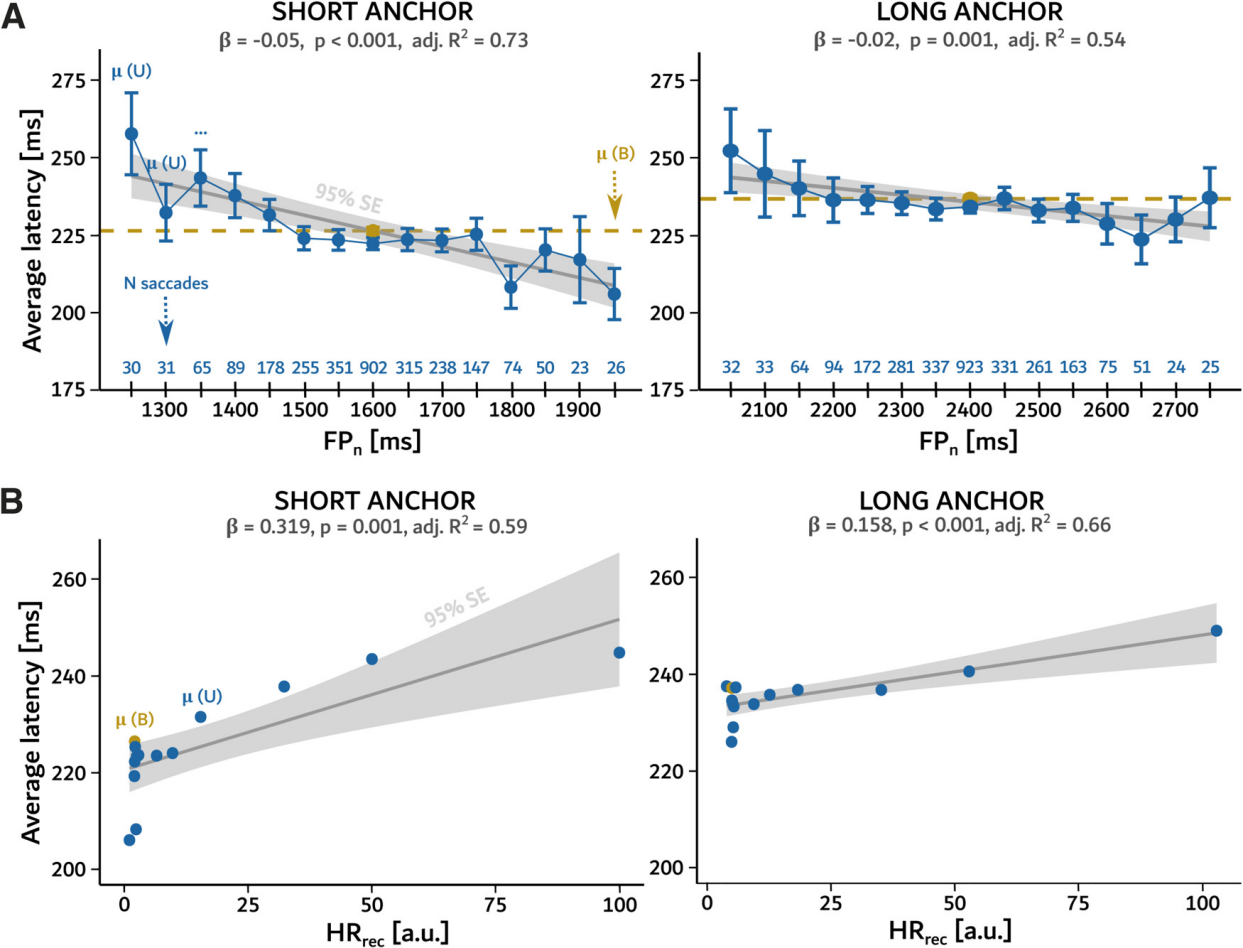
Analysis of saccade latencies as a function of FP duration. ***A***, Average saccade latency ± SE for different average FP durations. Each dot shows values for the corresponding bin of the distribution together with its SE (whiskers). Gray solid lines represent linear regression fits to the *U_short_* (left panel) and *U_long_* (right panel) distributions of the FP together with the 95% SE (gray ribbon). Yellow dotted lines indicate average latency for the B distributions. ***B***, Average saccade latency as a function of reciprocal HR (in a.u. - arbitrary units) for the *U_short_* (left panel) and *U_long_* (right panel) distributions.

The potential of the HR hypothesis to explain observed saccadic latencies was also tested with LMM. For both anchor durations, saccadic latency significantly decreased as a function of *HR_rec_* showing the effect of temporal preparation (model *HR_rec_.rs1*, short anchor: β = 0.371 ± 0.060, *p* = 8.86 × 10^–10^; long anchor: β = 0.190 ± 0.059, *p* = 0.001; see Table 3 for details). However, the relationship between reciprocal HR and RT had a shallower slope for the *U_long_* distribution (Fig. 2*B*).

**Table 3:** Linear mixed models analysis of *HR_rec_* effects on saccade latency

Dataset	Model	Fixed terms	β ± SE	95% CI	*p*-value	Random terms	σ [ms]
*U_short_*	*HR_rec_*.rs1	Intercept	217.924 ± 5.306	[207.398, 228.425]	<2.22 × 10^–16^	Residual	51.949
		*HR_rec_*	0.371 ± 0.060	[0.253, 0.489]	8.86 × 10^–10^	Subject	33.400
*U_long_*	*HR_rec_*.rs1	Intercept	231.100 ± 5.467	[220.272, 241.932]	<2.22 × 10^–16^	Residual	52.329
		*HR_rec_*	0.190 ± 0.059	[0.074, 0.306]	0.001	Subject	34.503

Models were fitted using the restricted maximum likelihood method.

σ, SD of the random terms (subject and residual).

10.1523/ENEURO.0496-22.2023.f2-1 Extended Data Figure 2-1 Plots of the equivalence test for the short (left panel) and the long anchor (right panel), separately. ***A***, Equivalence test results for the latency SD between different anchors in the U and B distribution. A black square shows mean difference in raw scores between B and U groups with the 98% confidence intervals (black horizontal line). ***B***, Equivalence test results for the *FP_n_*_–1_ effects on the saccade latency. Colored dot shows the mean effect size in standardized scores with the 98% confidence intervals (horizontal colored line). Download Figure 2-1, EPS file.

The fMTP model suggests that the short-term memory of previously experienced FPs (*FP_n_*_–1_) could partly determine saccadic latency during the current FP (*FP_n_*). Therefore, two possible memory effects were tested. First, there was no effect of the previous FP duration on the latency of the saccade in the current trial (*FP_n–1_.rs1* model: short anchor: *p* = 0.541; long anchor: *p* = 0.176; see Table 4 for details).

**Table 4:** Linear mixed models analysis of *FP_n_*_–1_ duration effects on saccade latency

Dataset	Model	Fixed terms	β ± SE	95% CI	*p*-value	Random terms	σ [ms]
*U_short_*	*FP_n_*_–1_.rs1	Intercept	213.039 ± 14.550	[184.560, 241.535]	<2.00 × 10^–16^	Residual	52.311
		*FP_n–1_*	0.005 ± 0.008	[−0.011, 0.022]	0.541	Subject	33.102
*U_long_*	*FP_n_*_–1_.rs1	Intercept	205.805 ± 20.649	[165.360, 246.253]	<2.00 × 10^–16^	Residual	52.410
		*FP_n–1_*	0.011 ± 0.008	[−0.005, 0.027]	0.176	Subject	34.389

Models were fitted using the restricted maximum likelihood method. For the equivalence tests related to the relationship between the saccade latency and FPn– 1 duration, see Extended Data Figure 2-1*B*.

σ, SD of the random terms (subject and residual).

Second, the sequence variable (difference between *FP_n_* and *FP_n_*_–1_) was fitted to the latency data. Saccadic latency significantly decreased as a function of sequence for both anchor durations (model *sequence.rs1*, short anchor: β = −0.029 ± 0.006, *p* = 7.01 × 10^–7^, long anchor: β = −0.020 ± 0.006, *p* = 8.41 × 10^–4^; see Table 5 for details).

**Table 5:** Linear mixed models analysis of sequence duration effects on saccade latency

Dataset	Model	Fixed terms	β ± SE	95% CI	*p*-value	Random terms	σ [ms]
*U_short_*	*sequence.rs1*	Intercept	220.861 ± 5.287	[210.372, 231.328]	<2.22 × 10^–16^	Residual	52.071
		Sequence	−0.029 ± 0.006	[−0.041, −0.018]	7.01 × 10^–7^	Subject	33.453
*U_long_*	*sequence.rs1*	Intercept	232.557 ± 5.442	[221.764, 243.332]	<2.22 × 10^–16^	Residual	52.321
		Sequence	−0.020 ± 0.006	[−0.031, −0.008]	8.41 × 10^–4^	Subject	34.498

Models were fitted using the restricted maximum likelihood method.

σ, SD of the random terms (subject and residual).

Beside the LMM analyses, a simple linear model (LM) was used to test specific predictions of the HR, PDF and fMTP models with latencies averaged across subjects and distinct FP lengths. Averaged saccadic latency decreased with increasing FP duration in *U_short_* (*p* = 3.01 × 10^–5^, adj. *R*^2^ = 0.73; Fig. 2*A*, left) and *U_long_* distribution (*p* = 0.001, adj. *R*^2^ = 0.54; Fig. 2*A*, right; see Table 6 for details). As expected, these results suggest that temporal preparation increased during the FP. Furthermore, a linear relationship between average latency and *HR_rec_* was found for the *U_short_* (*p* = 0.001, adj. *R*^2^ = 0.59) and *U_long_* distribution of FPs (*p* = 5 × 10^–4^, adj. *R*^2^ = 0.66; see Table 6 for details). However, no local minima were observed around anchor FP durations.

**Table 6:** Linear models and slope analysis (Ivry and Hazeltine, 1995) **results for saccade latency**

Dataset	Formula	β ± SE	95% CI	*p*-value	Adj. *R*^2^
*U_short_*	Latency $∼FP_{n}$	−0.051 ± 0.008	[−0.068, −0.033]	3.01 × 10^–5^	0.73
	Latency $∼HR_{rec}$	0.319 ± 0.074	[0.155, 0.484]	0.001	0.59
	σ^2^ (latency) $∼k^{2}$* $FP_{n}^{2}$ *+ c*	−18704.922 ± 7683.918	[−35305.020, −2104.826]	0.030	0.26
	σ (latency) $∼HR_{classic}$	−23.317 ± 3.831	[−31.748, −14.885]	7.88 × 10^–5^	0.75
*U_long_*	Latency $∼FP_{n}$	−0.023 ± 0.005	[−0.035, −0.011]	0.001	0.54
	Latency $∼HR_{rec}$	0.158 ± 0.032	[0.087, 0.228]	5 × 10^–4^	0.66
	σ^2^ (latency) $∼k^{2}$ * $FP_{n}^{2}$ *+ c*	−42253.758 ± 7100.721	[−57593.93, −26913.580]	4.82 × 10^–5^	0.71
	σ (latency) $∼HR_{classic}$	−22.625 ± 5.603	[−34.957, −10.293]	0.002	0.56

The dataset was averaged across subjects and distinct *FP_n_* lengths.

σ^2^, variance; *k*, Weber’s fraction; *c*, time-independent source of response variance; adj., adjusted.

**Figure 3.**
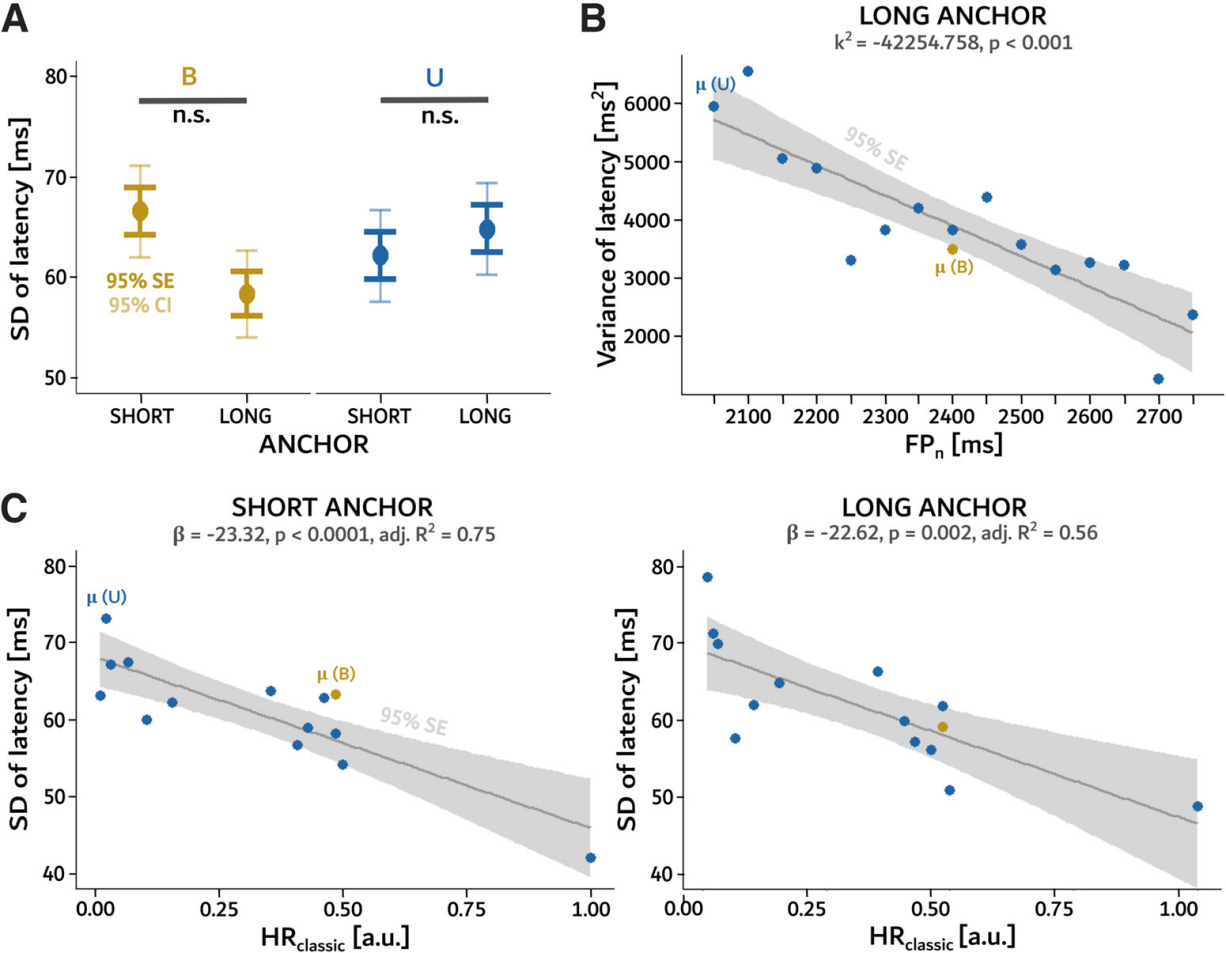
Analysis of the variability of saccadic latencies. ***A***, SD of saccade latency in the different experimental conditions (dots) together with its SE (dark whiskers), and 95% confidence interval (CI, light whiskers). Horizontal gray bars indicate paired analysis between conditions (n.s., not significant). For the equivalence tests related to the magnitude of difference between the SD of the saccade latency, see Extended Data Figure 2-1*A*. ***B***, Variance of saccadic latencies plotted against different FP lengths. Each dot shows the value for the corresponding bin of the U distributions. The solid line shows a linear regression line together with the standard error of the regression SE (gray ribbon). Yellow dot indicates average variance around anchor durations. ***C***, SD of saccade latencies plotted against *HR_classic_* for different FP lengths. Each dot shows the value for the corresponding bin of the U distributions. Solid lines show linear regression lines together with the standard error of the regression SE (gray ribbon). Yellow dot indicates average SDs around anchor durations.

The HR, PDF and fMTP models all suggest that the SD of timing responses should increase with anchor because of temporal “blurring” or scalar expectancy. However, in the present study, the SD of saccadic latency for long and short anchors did not increase (B conditions, *t*_(41)_ = 0.663, *p* = 0.511; U conditions, *t*_(41)_ = 0.060, *p* = 0.952; see Fig. 3*A* for details). Furthermore, the SD of saccade latencies was also computed when movements were grouped according to the bins of the U distributions. To test the relationship between the SD of the latency and the length of the FP in the given trial, a slope analysis was conducted. The SD of saccadic latencies significantly decreased with elapsed time for the *U_long_* condition (*p* = 3.84 × 10^–5^, adj. *R*^2^ = 0.72; see Table 6). However, as the square of the Weber’s fraction was negative (*k*^2^ = −42 253.758), Weber’s law was not obeyed and the scalar property was not detected. Instead, the relationship between the SD of the saccadic latency distributions and variants of the hazard rate hypothesis was tested. Unexpectedly, the SD significantly decreased with *HR_classic_* for both anchors (*p* = 7.88 × 10^–5^, adj. *R*^2^ = 0.75 for short and *p* = 0.002, adj. *R*^2^ = 0.56 for long anchor; see Table 6 and Fig. 3*B* for details) and therefore presented a better fit than *FP_n_*.

Equivalence tests were used to check whether the effect size obtained for the nonsignificant analyses was small enough to be called negligible (see Lakens et al., 2018 for details). Both equivalence tests related to the magnitude of difference between the SD of the latency in *B_short_* and *B_long_* condition (*t*_(41)_ = 5.330, *p* = 2.00 × 10^–6^) as well as *U_short_* and *U_long_* conditions were significant (*t*_(41)_ = 3.254, *p* = 0.001; see Extended Data Fig. 2-1*A*). Therefore, in both those cases, the null equivalence hypothesis could be rejected and the size of the effect for both analyses was negligible. Finally, the equivalence test of the influence of the FP(*n*−1) length on the saccadic latency was shown significant for the short anchor (*p* < 2.22 × 10^–16^, 98% CI = [−0.01, 0.02]), but the test for the long anchor was not conclusive (*p* = 0.145, 98% CI = [−0.01, 0.03], see Extended Data Fig. 2-1*B*). Therefore, the null equivalence hypothesis was rejected and the negligible character of the effect size was accepted only for the short anchor.

## Discussion

The aim of the present study was to investigate the temporal “blurring” hypothesis by altering stimulus-bound and subjective uncertainties in an oculomotor reaction time task. Subjects were firstly tested in a baseline condition B with a delay between the warning stimulus (WS) and an imperative “go” signal that was kept constant, either 1600 ms (short anchor) or 2400 ms (long anchor). One hundred B trials with one of these two anchor (*B_short_* or *B_long_*) durations were firstly collected. Afterwards, stimulus-bound temporal uncertainty was increased and 100 trials from the U distribution were presented (same anchor duration as in B but σ = 120 ms; see Fig. 1 and Materials and Methods for more details). After data collection using both B and U distributions and the first anchor duration, the whole process was repeated with the other anchor duration. The dependent variable measured was saccadic latency.

First, a scalar increase of the variance of latencies between short (*B_short_*) and long (*B_long_*) anchor durations (Fig. 3*A*) was not observed. This suggests that scalar expectancy was not observed in the present results. Second, comparison of U distributions revealed that the slope of the saccadic latency/FP relationship was shallower for long anchors (*U_long_*) than for short ones (*U_short_*; see Fig. 2*A* and Tables 2, 6 for details). These results suggest a reduced sensitivity to time around the long anchor duration. Third, no latency minimum around the anchor values was observed (Fig. 2*A*). This result suggests that latency was not directly dependent on FP probability. Fourthly, saccadic latencies did not co-vary with FP duration in the 1500- to 1750-ms range in the *U_short_* condition and in the 2100- to 2550-ms range in the *U_long_* condition (Fig. 2*A*). Subjectively, FP durations in these indifference ranges could have been interpreted as equivalent to the two anchor durations. The indifference range was larger for long durations, suggesting again an impact of subjective uncertainty but not as predicted by the temporal blurring hypothesis nor the PDF hypothesis. Therefore, scalar expectancy *stricto sensu* was not observed when comparing between distributions, neither in the B nor in the U conditions. Within U distributions, the variability of latencies unexpectedly decreased with increasing FP durations (see Table 6). The gradual decrease of the variability of movement latency could be modelled using the classic HR function (see Table 6). In summary, HR could have a double impact: reducing movement latency and increasing precision.

It has been suggested that scalar expectancy could not be observed in implicit oculomotor timing (Ameqrane et al., 2014). Scalar property would be present only in explicit timing tasks when there is an overt use of temporal information (Nobre and van Ede, 2018; Coull and Nobre, 2008). However, Piras and Coull (2011) suggested that scalar variability was indeed observed in a temporal expectancy task in humans. How could the volatility of scalar expectancy be explained? The oculomotor variant of scalar expectancy was initially proposed by Janssen and Shadlen (2005) in the Rhesus monkey where intensive animal training and reward expectation could have led to the emergence of an explicit representation of elapsed time during the FP, like in prospective timing. Moreover, it could be suggested that the motor system used to provide a response could also play a major role. Indeed, to our knowledge, most human timing studies required a manual response (typically a button press) after a visual or auditory “go” signal was presented (Piras and Coull, 2011; Grabenhorst et al., 2019, 2021; Tomassini et al., 2019; Visalli et al., 2021; Tal-Perry and Yuval-Greenberg, 2022; but for critical review, see Grondin, 2012). This could have induced another sensorimotor mapping with explicit attention devoted to the production of an accurate manual response, therefore scalar expectancy. A systematic comparison between manual and oculomotor responses over the same range of durations and for the same probability distributions remains to be done.

**Figure 4.**
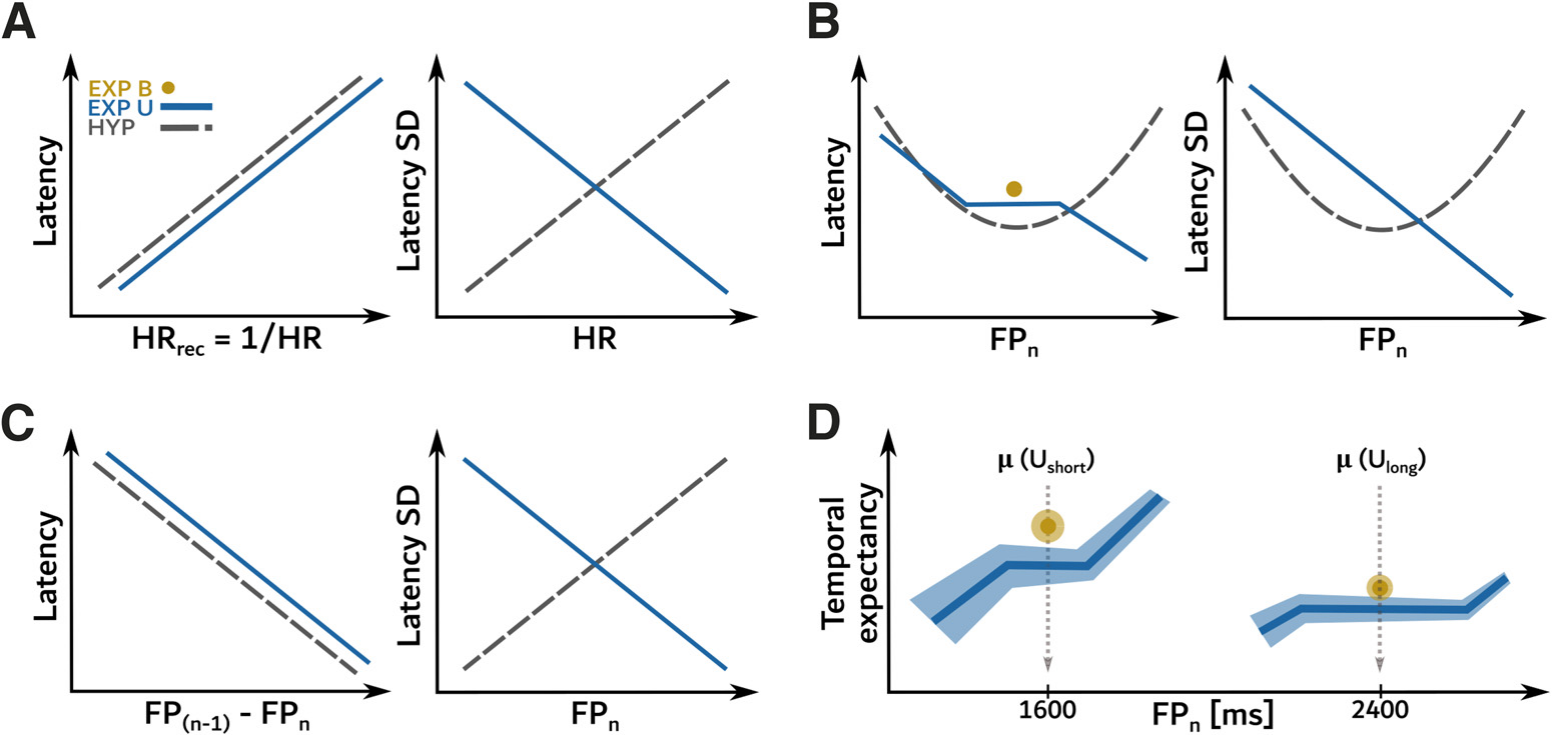
Comparison of dominant hypotheses of temporal preparation and empirical data. Predictions of temporal preparation models (gray dotted curves) versus experimental data (blue lines for the uncertainty U condition and yellow dots for baseline B condition) for average latency (left panels) and SD (right panels). ***A***, Left, Saccadic latency increased as a function of reciprocal of HR (*HR_rec_*). Right, The SD of saccadic latency decreased with increasing FP duration, in contradiction with the temporal blurring hypothesis based on HR. ***B***, Left, Saccadic latency decreased with FP duration in contradiction with predictions of the PDF hypothesis. Indeed, where the PDF is maximum, movement latency should be minimum and “go” signal timings infrequently experienced on the sides of the PDF should evoke longer latencies (inverted U-shape). Right, Probabilistic “blurring” hypothesis. Movement timing variability should follow an inverted U-shape distribution. ***C***, Left, As predicted by the fMTP model, a shorter FP duration during trial “*n*−1” than during the current trial “*n*” is associated with a relatively shorter movement latency. Right, The fMTP model assumes temporal blurring of the inner representation of FP duration and an increasing saccadic SD for longer FP durations. This is functionally equivalent to the one assumed by the HR hypothesis and was not observed here. ***D***, Hypothetical build up of temporal expectancy of the imperative “go” signal for short and long anchor durations.

It could also be suggested that scalar expectancy was not observed in the present study because temporal preparation itself was reduced. However, a robust reduction of movement latency with increasing FP duration in the U conditions was shown in the current research. Figure 4 shows a schematic comparison of predictions issued from current hypotheses about temporal preparation (dashed lines) and experimental results presented here (blue continuous lines and yellow dots). If the PDF hypothesis were true then the latency/FP relationship should be U-shaped with a minimum around the mean where “go” signal timing was the most frequent (Fig. 4*A*; see Grabenhorst et al., 2019, 2021). This was not observed. If temporal preparation was based on an estimation of the objective (*HR_classic_*) or subjective (*HR_subj_*) hazard rate (Fig. 4*B*) then shorter latencies should be observed for long FP durations where these functions reached a maximum. This prediction was confirmed within U distributions. However, the *HR_subj_* hypothesis additionally implies that the variance of latencies should increase with elapsed time. This was not observed here. Altogether, the best model fit to the average latency data were the reciprocal of HR (*HR_rec_*).

Two memory-related effects were tested in the current study. A memory effect of the previously experienced FP duration (*FP_n_*_–1_) on saccade latency was not observed here. This is in contradiction with previously published studies from authors group that demonstrated a strong influence of trial history on the latency of saccades during trial “*n*” even if the total number of trials was rather small (∼100–200 trials; see Ameqrane et al., 2014; Degos et al., 2018; Hsu et al., 2019). Authors suggest that the memory of past FPs could play a role only if the total number of possible “go” signal timings is small. On the other hand, a sequence effect was found here (*difference* between *FP_n_* and *FP_n_*_–1_) as suggested by the fMTP model (Visalli et al., 2021; Salet et al., 2022). Nevertheless, temporal blurring was also hypothesized in the fMTP model, in contradiction with the observation in the current study. In conclusion, the hazard rate hypothesis provides a better explanation of the empirical data than other approaches. The *HR_rec_* function could be used to model the influence of temporal preparation on average latency whereas the *HR_classic_* function could model the reduction of movement variability with elapsed time. These results suggest that temporal blurring does not necessarily occur and that the particular timing context, implicit or explicit, plays a major role.

## Synthesis

Reviewing Editor: Niko Busch, Westfalische Wilhelms-Universitat Munster Decisions are customarily a result of the Reviewing Editor and the peer reviewers coming together and discussing their recommendations until a consensus is reached. When revisions are invited, a fact-based synthesis statement explaining their decision and outlining what is needed to prepare a revision will be listed below. The following reviewer(s) agreed to reveal their identity: Sophie Herbst. Note: If this manuscript was transferred from JNeurosci and a decision was made to accept the manuscript without peer review, a brief statement to this effect will instead be what is listed below.

# Synthesis

The revised manuscript has been reviewed again by two experts on temporal expectations (one of them was also a reviewer of the previous submission). Both reviews appreciate the substantial revisions and improvements to the manuscript. Specifically, reviewer 1 recommends further effort to make the manuscript of accessible, and I strongly sympathize with this request. Moreover, reviewer 2 has several concerns regarding the statistical analyses. I encourage you to carefully address each of these points in your revision.

# Reviewer 1

Thank you for the thorough revision and for addressing the previous comments. The manuscript has improved a lot in clarity.

My main issue is that I still found it very difficult to understand the results section. The following suggestions might help:

- Move all explanations and motivation for the analyses to the methods section

- Follow exactly the same order of reporting in Methods and Results

- Separate the model selection process from the reporting of the actual results, for instance in two separate sections

- Explain the naming scheme for the models in the paper (I understood it from the reply letter, but in the paper it was not clear)

In the same respect:

Please clarify the distinction between the analyses described in lines 162 - 172 and Table 3, versus 173 - 177 and Table 4:

Table 3: What is the unity of the τ (tau) values reported in the last column in Table 3 (and 5), and why are they so big? The caption below says ‘τ variance of the random terms’, but especially for a variance, these values are very high, and variances are not necessarily integers.

The summary of results on p. 12, l. 240 and follows is very helpful. Can you provide links to the Figures and Tables that show these results?

Add panel headings and color legends to Figure 4.

Methods:

I found these side-by-side statements confusing, even though it becomes clear later in the text. Rephrase to avoid apparent mismatch?

l. 91: After a variable delay (foreperiod or ‘FP’, lasting between 1250 and 2750 ms) the 91

imperative ‘go’ stimulus (‘IS’, eccentric red square) was displayed in the eccentric box for 50 ms.

l. 96: In each block of trials, average FP duration was either 1600 ms (short) or 2400 ms (long), collectively referred to as ‘FPdur’.

# Reviewer 2

I was approached by the editor to go over the first reviewer’s comments and the authors’ rebuttal thereof, in lieu of the lack of the first reviewer’s availability. To get the full picture, I first read the manuscript in its revised form and then proceeded to inspect the first reviewer’s comments.

In this study, the authors tested the assumption that we are less able to predict the onset of more distant future events than proximal events, an assumption referred to as “temporal blurring” and related to the scalar expectancy - the linear increase in estimation variance as temporal distance increases. This was examined via an implicit timing task (a WS-S task) using oculomotor response, with the interval between the cue and the target drawn from a short or long interval distribution. The main finding was a lack of increase in saccade latency variance between the two distributions, in contrast to the temporal blurring prediction.

I find the research question to be of interest to researchers within the field. The manuscript itself is well-worded, the experimental design is straightforward and suitable to answer the research question, and the sample size and the number of trials performed per participant seem adequate to observe a conclusive result.

From the authors’ rebuttal to the first reviewer’s comments, I understand the previous version of this manuscript included a pupil size analysis, that was decided to be excluded from the current version of the paper. Thus, comments 1 and 2 made by the reviewer are no longer relevant, so I will ignore them.

In the third comment, the reviewer suggested including the trial number as a covariate in the model predicting saccade latency. I find this recommendation sensible (in alignment with Baayen & Milin, 2010), and would recommend the same. The authors responded that adding the variable as a covariate did not improve model fit, and thus this approach was abandoned. While that is okay, I would suggest mentioning it within the manuscript and adding that to the supplementary data (or Extended Data relating to the relevant table), since I could imagine the same reasoning might occur to other readers.

Besides the major points, the first reviewer also made two minor points. The first of which is no longer relevant in the current version of the manuscript, so I will ignore it. The second point is related to the place of the final figure. The reviewer suggested the figure is more suitable in the Results section, while the authors believe it should be within the Discussion, arguing that the paper was not intended to compare the models. As the reviewer mentioned, I think it’s a matter of personal taste. I for once believe the figure should actually be presented at the end of the introduction to summarize and visualize the different models discussed. The results of the papers could then be added in brief at the end of the introduction section, such that the figure could remain as is. However, I leave this to the discretion of the editor or to the authors’ preference.

Having covered that, while reading the manuscript I encountered several major issues not raised by the reviewer, possibly due to changes made to the manuscript. Since these issues relate directly to the main conclusions made by the authors, I am sorry to say that these should probably be addressed in order to make a compelling argument.

1) I have some concerns regarding the authors’ choice of statistical analysis. The authors implemented a linear mixed model (LMM) approach to analyze saccade latency. However, saccade latency is known to have a skewed distribution, just like reaction time (e.g., Summer, 2011). Thus, the normality of residuals assumption is likely violated here, potentially leading to a misestimation of error terms. Although the authors mention they performed model diagnostics to check for possible deviations from model assumptions (lines 138-140), I believe the data type analysed here requires more compelling evidence to indicate that. Minimally, I would suggest the authors defend their choice of LMM with supporting evidence (in supplementary data or Extended data), such as QQ plots or model fits to normal vs exponential families. The R package ‘performance’ (Lüdecke et al., 2021) provides an easy solution to do so (using the check_normality and check_distribution functions). Alternatively, the authors could perform a generalized linear mixed model (GLMM) using gamma or possibly an inverse-gaussian family of responses (see Baayen & Milin, 2010; Lo & Andrews, 2015), or possibly transform the raw saccade latencies into a measurement that is adequately normally distributed (while additionally supplying supporting evidence for that).

2) The above concern is amplified for the SD analysis depicted in Table 4 and lines 217-222. The distribution of SD is typically gamma shaped and is bounded by zero, and thus it cannot be normally distributed. It is possible that the authors can implement a generalized linear model with a gamma family of response to analyze the SD or variance component as a function of a continuous variable, although I admit I have not seen or performed a similar analysis. It is possible a slope analysis (as in Ameqrane et al., 2014; Piras & Coull, 2011) is more fitting to analyse these results.

3) I am also unsure about the modelling of the previous foreperiod on the current saccade latency. For starters, it is not clear to me what exactly was used within the model - the actual foreperiod, or the difference between the current and previous foreperiod. The sequential effect, as captured by the fMTP model, suggests that the difference between the current and the previous foreperiods is what influences anticipation - a matching in foreperiod leads to a high expectation at the time of stimulus onset, while a mismatch (predominantly, previous shorter than current) leads to low expectations at the time of stimulus onset, which leads to slower reaction time. If the results were modelled this way, I suggest revising the wording to make that clearer. If not, I am not sure the current results support the authors’ claim, as they do not capture the sequential effect as described in the fMTP model, and I would suggest the authors use the difference in foreperiod as a fixed continuous variable. Additionally, it is unclear to me why the authors chose to model the FPn-1 effect as a separate model, excluding the effect of the current FP as modelled in Table 3.

4) Finally, two arguments the authors make in the discussion are based on null results, namely the lack of scalar increase in variance between the short and long anchors, and the lack of memory effect for previously experienced foreperiod. While I raised some concerns regarding the results of these two analyses, I would also like to point out that the authors should be wary of drawing support for the null hypothesis from a lack of significant results (e.g., Parkhurst 2001). In case a null result is again observed in the new analyses I’ve suggested above, I would recommend performing an equivalent test (Lakens et al., 2018; also, Parkhurst 2001), in case the authors wish to remain within the frequentist framework, or alternatively, implement Bayesian statistics to calculate Bayes Factor in favour of the null hypothesis (e.g., Rouder et al., 2009). Since the authors already calculated BIC for the majority of their analyses, a Bayes Factor could easily be approximated from the difference in BIC between models (ΔBIC; see Jarosz & Wileyand, 2014).

Since at least some of the main conclusions of the authors rest on these results (importantly, the lack of change in scalar variance as tested by the difference in variance latencies, but also the validity of HR hypothesis as modelled in LMM and the refutation of the fMTP model), I am afraid I cannot recommend the manuscript be accepted in its current form.

Minor issues:

1) I find the procedure to be missing some important information, specifically the participant’s sitting distance from the screen, and the screen size (normally specified diagonally in inch, or width X height in cm). As these parameters affect the saccade amplitude and velocity calculations, which are the parameters used for saccade detection criteria, I believe they ought to be specified.

2) The authors mentioned that only saccades with amplitude above 1 visual degree were included in the analysis. However, since the eccentric box was displayed at a 9.5 visual eccentricity, I was wondering whether this criterion might include spurious saccades that did not actually land on the target box. I believe it might be sensible to add another spatial criterion on the saccade landing position. However, I consider it a minor point since I do not believe it should affect results that much.

3) I believe there are a few studies relevant to some of the points made in the discussion that are worth including. The authors mention that the scalar property is observed only for explicit timing tasks, i.e., tasks in which time is overtly attended. However, there are works that also demonstrate this scalar property for implicit timing tasks, i.e., tasks in which time is used in order to attend to a future event (e.g., Piras & Coull, 2011). Later in the same paragraph, the authors mention the difference between manual (hand) and oculomotor movement and the relation to scalar expectancy. There is a study on this subject for oculomotor movement (Ameqrane et al., 2014, mentioned elsewhere in the manuscript) whose results are probably worth being mentioned in this discussion.

4) Line 128: 5,07% should probably read as 5.07%

References

Ameqrane, I., Pouget, P., Wattiez, N., Carpenter, R., & Missal, M. (2014). Implicit and Explicit Timing in Oculomotor Control. PLOS ONE, 9(4), e93958. https://doi.org/10.1371/journal.pone.0093958

Baayen, H. R., & Milin, P. (2010). Analyzing reaction times. International Journal of Psychological Research, 3(2), 12-28. https://doi.org/10.21500/20112084.807

Lakens, D., Scheel, A. M., & Isager, P. M. (2018). Equivalence Testing for Psychological Research: A Tutorial. Advances in Methods and Practices in Psychological Science, 1(2), 259-269. https://doi.org/10.1177/2515245918770963

Jarosz, A. F., & Wileyand, J. (2014). What Are the Odds? A Practical Guide to Computing and Reporting Bayes Factors. Journal of Problem Solving, 7. https://doi.org/10.7771/1932-6246.1167

Lo, S., & Andrews, S. (2015). To transform or not to transform: Using generalized linear mixed models to analyse reaction time data. Frontiers in Psychology, 6(August), 1-16. https://doi.org/10.3389/fpsyg.2015.01171

Lüdecke D, Ben-Shachar M, Patil I, Waggoner P, Makowski D (2021). “performance: An R Package for Assessment, Comparison and Testing of Statistical Models.” Journal of Open Source Software, 6(60), 3139. doi:10.21105/joss.03139.

Parkhurst, D. F. (2001). Statistical Significance Tests: Equivalence and Reverse Tests Should Reduce Misinterpretation: Equivalence tests improve the logic of significance testing when demonstrating similarity is important, and reverse tests can help show that failure to reject a null hypothesis does not support that hypothesis. BioScience, 51(12), 1051-1057. https://doi.org/10.1641/0006-3568(2001)051[1051:SSTEAR]2.0.CO;2

Piras, F., & Coull, J. T. (2011). Implicit, predictive timing draws upon the same scalar representation of time as explicit timing. PLoS ONE, 6(3). https://doi.org/10.1371/journal.pone.0018203

Rouder, J. N., Speckman, P. L., Sun, D., Morey, R. D., & Iverson, G. (2009). Bayesian t tests for accepting and rejecting the null hypothesis. Psychonomic Bulletin and Review, 16(2), 225-237. https://doi.org/10.3758/PBR.16.2.225

Sumner, P. (2011). Determinants of saccade latency. The Oxford handbook of eye movements, 413-424.
