# Supplementary material for: How Does Temporal Blurring Alter Movement Timing?
Source: eNeuro. 2023 Sep 11;10(9):ENEURO.0496-22.2023. doi: 10.1523/ENEURO.0496-22.2023 (PMC10500974; doi:10.1523/ENEURO.0496-22.2023)
Supplement: Table 1-1 — Linear mixed models random structure selection for the analysis of FPn, FPn-1 HRrec and sequence effects on saccade latency. Models were fitted using the restricted maximum likelihood method. Download Table 1-1, DOC file. [file enu-eN-NWR-0496-22-s04.doc]

**Table 1-1.** **Linear mixed models random structure selection for the analysis of FP_n_, FP_n−1_ HR_rec_ and sequence effects on saccade latency.** Models were fitted using the restricted maximum likelihood method.

| Main predictor | Model | Formula | BIC | |
| --- | --- | --- | --- | --- |
|  |  |  | U_short_ | U_long_ |
| FP_n_ | full.rs1 | latency ∼ FP_n_*trial + (1 | id) | 29980.299 | 31024.170 |
|  | full.rs2 | latency ∼ FP_n_*trial + (1 + FP_n_ \| id) | 29996.329 | 31035.453 |
|  | full.rs3 | latency ∼ FP_n_*trial + (1 + trial \| id) | 29995.063 | 31038.805 |
|  | full.rs4 | latency ∼ FP_n_*trial + (1 + FP_n_*trial \| id) | 30978.917 | 32065.616 |
| HR_rec_ | full.rs1 | latency ∼ HR_rec_*trial + (1 | id) | 29977.343 | 31017.092 |
|  | full.rs2 | latency ∼ HR_rec_*trial + (1 + HR_rec_ \| id) | 29986.400 | 31030.359 |
|  | full.rs3 | latency ∼ HR_rec_*trial + (1 + trial \| id) | 29992.063 | 31031.710 |
|  | full.rs4 | latency ∼ HR_rec_*trial + (1 + HR_rec_*trial \| id) | 30040.494 | 31321.244 |
| FP_n-1_ | full.rs1 | latency ∼ FP_n-1_*trial + (1 | id) | 30022.660 | 31033.178 |
|  | full.rs2 | latency ∼ FP_n-1_*trial + (1 + FP_n-1_ \| id) | 30037.740 | 31045.446 |
|  | full.rs3 | latency ∼ FP_n-1_*trial + (1 + trial \| id) | 30037.477 | 31047.792 |
|  | full.rs4 | latency ∼ FP_n-1_*trial + (1 + FP_n-1_*trial \| id) | 31006.892 | 32090.149 |
| sequence | full.rs1 | latency ∼ sequence*trial + (1 | id) | 29999.334 | 31025.092 |
|  | full.rs2 | latency ∼ sequence*trial + (1 + sequence \| id) | 30013.897 | 31040.940 |
|  | full.rs3 | latency ∼ sequence*trial + (1 + trial \| id) | 30014.136 | 31039.662 |
|  | full.rs4 | latency ∼ sequence*trial + (1 + sequence*trial \| id) | 30347.758 | 31499.177 |

*BIC* Bayes Information Criterion, *full* maximal fixed terms structure, *rs* random structure
