# Supplementary material for: How Does Temporal Blurring Alter Movement Timing?
Source: eNeuro. 2023 Sep 11;10(9):ENEURO.0496-22.2023. doi: 10.1523/ENEURO.0496-22.2023 (PMC10500974; doi:10.1523/ENEURO.0496-22.2023)
Supplement: Table 1-2 — Linear mixed models random structure selection for the analysis of FPn, FPn-1 HRrec and sequence effects on saccade latency. Models were fitted using the restricted maximum likelihood method. Download Table 1-2, DOC file. [file enu-eN-NWR-0496-22-s05.doc]

**Table 1-2.** **Linear mixed models fixed structure selection for the analysis of FP_n_, FP_n−1_ HR_rec_ and sequence effects on saccade latency.** Models were fitted using the maximum likelihood method.

| Main predictor | Model | Formula | BIC | |
| --- | --- | --- | --- | --- |
|  |  |  | U_short_ | U_long_ |
| FP_n_ | null.rs1 | latency ∼ 1 + (1 | id) | 29983.567 | 30989.858 |
|  | FP_n_.rs1 | latency ∼ FP_n_ + (1 \| id) | 29949.710 | 30986.900 |
|  | full.rs1 | latency ∼ FP_n_*trial + (1 \| id) | 29958.363 | 31002.218 |
| HR_rec_ | null.rs1 | latency ∼ 1 + (1 | id) | 29983.567 | 30989.858 |
|  | HR_rec_.rs1 | latency ∼ HR_rec_ + (1 \| id) | 29953.948 | 30987.479 |
|  | full.rs1 | latency ∼ HR_rec_*trial + (1 \| id) | 29963.295 | 31003.072 |
| FP_n-1_ | null.rs1 | latency ∼ 1 + (1 | id) | 29983.567 | 30989.858 |
|  | FP_n-1_.rs1 | latency ∼ FP_n-1_ + (1 \| id) | 29991.123 | 30995.986 |
|  | full.rs1 | latency ∼ FP_n-1_*trial + (1 \| id) | 30000.808 | 31011.265 |
| sequence | null.rs1 | latency ∼ 1 + (1 | id) | 29983.567 | 30989.858 |
|  | sequence.rs1 | latency ∼ sequence + (1 \| id) | 29966.906 | 30986.671 |
|  | full.rs1 | latency ∼ sequence*trial + (1 \| id) | 31011.265 | 31011.265 |

*BIC* Bayes Information Criterion, *full* maximal fixed terms structure, *null* no fixed terms structure, *rs* random structure
